# Comparison of the clinical efficacy of two fixation methods combined with OLIF in the treatment of lumbar spondylolisthesis in adult patients

**DOI:** 10.1186/s13018-022-02991-z

**Published:** 2022-02-21

**Authors:** Xinliang Zhang, Yunshan Guo, Yibing Li

**Affiliations:** Department of Spine Surgery, Xi’an Jiaotong University Affiliated Honghui Hospital, Xi’an, 710000 Shaanxi China

**Keywords:** OLIF, Anterior approach, Single rod-screw, Percutaneous pedicle screw

## Abstract

**Background:**

To observe the clinical efficacy of an anterior single rob-screw fixation (ASRSF) combined with the oblique lumbar intervertebral fusion (OLIF) approach compared with a posterior percutaneous screw fixation (PPSF) combined with OLIF in the treatment of lumbar spondylolisthesis.

**Method:**

This is a retrospective case–control study. Patients with degenerative lumbar spondylolisthesis (DLS) treated with either ASRSF combined with OLIF or PPSF combined with OLIF from January 2016 to January 2018 were enrolled in this study. None of the patients had posterior decompression. The visual analog scale (VAS) and Oswestry dysfunction index (ODI) were used for clinical efficacy assessment. The pre- and post-operational disc height, height of foramen, subsidence, and migration of cages, fusion rate and surgery-related complications were compared between the two groups.

**Results:**

Fifty-three patients were included in this single-center study. According to the fixation methods, patients were divided into the ASRSF group (group A, 25 cases) and the PPSF group (group B, 28 cases). There was no statistical difference in surgery-related complications between groups. There was a significant difference in the VAS score at one-week post-surgery (2.3 ± 0.5 vs. 3.5 ± 0.4, *P* = 0.01), and three months post-operation (2.2 ± 0.3 vs. 3.0 ± 0.3, *P* = 0.01). Comparison of post-operative imaging data showed that there was a significant difference in the height of the foramen between groups at three months post-surgery(18.1 ± 2.3 mm vs. 16.9 ± 1.9 mm, *P* = 0.04). At 24 months post-surgery, the ODI was 12.65 ± 3.6 in group A and 19.1 ± 3.4 in group B (*P* = 0.01). Twelve months after surgery, the fusion rate in group A at 72.0% and 78.6% in group B was not statistically significant (*P* = 0.75). Fusion was identified in all patients at 24 months post-surgery.

**Conclusion:**

When compared to PPSF, ASRSF combined with OLIF for DLS can reduce post-operative low back pain in the initial stages, maintain the height of the foramen and improve the performance of lumbar function.

## Background

With the aging population, degenerative spinal disease (DSD) has become an important pathological cause of immobility and incapacitation in elder adults. Globally, the incidence of DSD exceeds 43.1%, of which 35.3% have severe dysfunction [1–4]. The incidence of DSD in low- and middle-income classes is four times higher than that of the high-income class and has increasingly become a social issue that seriously affects the quality of life of many people [2, 3, 5]. Among DSD patients, the incidence of degenerative lumbar spondylolisthesis (DLS) is more than 12.5% [2, 3] and pathologies are complicated. It most commonly affects the lower lumbar spine, although it has been reported in the cervical and thoracic spine secondary to trauma. Disc degeneration and the subsequent narrowness of intervertebral space may lead to the final slippage[3, 4]. The reported prevalence ranges from 19.1 to 43.1% with a mean age ranging from 71.5 to 75.7 years and a higher female preponderance [6].

After the failure of conservative treatments, patients with DLS often require surgery. The primary surgical goal is decompression and long-term stability in the target segments. Fusion is typically the most efficient treatment in this type of surgery and current methods include transforaminal lumbar interbody fusion (TLIF), anterior lumbar interbody fusion (ALIF), posterior lumbar interbody fusion (PLIF) and oblique lumbar intervertebral fusion (OLIF). The OLIF technology has attracted attention from surgeons due to its advantages in reducing trauma, improving safety, and restoring lumbar lordosis. With the recent application of the standalone cage, the surgical procedure has been simplified but had uncertain post-operative stability [6]. Considering early post-operative stability, OLIF often requires combined posterior fixation with iatrogenic soft tissue impairment. From the perspective of soft tissue protection, OLIF combined with posterior percutaneous screws has become a mainstream procedure having superior stability and better clinical outcomes [7–10], but due to the lack of anatomical landmarks, the risk of screw misplacement and nerve damage is high [7, 8].

Anterior single screw fixation is commonly used in anterior surgery for deformity correction and debridement. This treatment has shown excellent performance in maintaining stability and reducing iatrogenic trauma and it has been speculated that this treatment could provide additional stability in the OLIF procedure. The present study aims to analyze the clinical efficacy of the anterior single screw rod combined with OLIF compared to using a posterior percutaneous pedicle screw combined with OLIF in the treatment of lumbar spondylolisthesis. It is hypothesized that anterior fixation will significantly reduce post-surgery pain, by avoiding the paraspinal muscle.

## Materials and methods

### Study design

This retrospective case–control study included patients who underwent OLIF surgery for DSD from January 2016 to January 2018 in Xi'an Jiaotong University Affiliated Honghui hospital medical center. This study was approved by the Ethics Committee of Xi'an Jiaotong University (approval number: 201606012). Given the retrospective nature of the study, patients’ informed consents were not necessary.

The diagnosis of spondylolisthesis was based on pre-operative X-ray examination. Inclusion criteria include imaging confirmed lumbar spondylolisthesis located between lumbar vertebrae two to four, clinical symptoms related to spondylolisthesis, such as low back pain and claudication, four to six weeks of conservative treatment including brace fixation and medications without symptom alleviation, degree of slippage classified as Meyerding Grade I or II and patient follow-up for more than 24 months. Exclusion criteria were previous history of lumbar spine surgery, post-operative residual symptoms that required secondary direct decompression, incomplete medical data records and lumbar isthmic spondylolisthesis. Symptoms for surgery included refractory low back pain and claudication with negative reaction from conservative treatments including medication and physical therapies.

### Surgical procedures

All operations were performed by the corresponding author. Left side surgical approaches were used in all cases. The presence of scoliosis did not affect the side of the surgical approach. After a five-cm skin incision was made, six to ten cm anterior to the mid-portion of the marked disc, the surgeon approached the retroperitoneal space by blunt dissection and mobilization of the peritoneum anteriorly to expose the anatomical oblique lateral corridor. The soft tissue was expanded, then the working channel was placed and if necessary, the segmental blood vessels were ligated. After discectomy and endplate preparation, the appropriate cage (Clydesdale spinal system, Medtronic, Memphis, TN,USA) 12 mm in height, 50 mm in length and 18 mm in width, classed as 6° lordotic, made of polyether ether ketone and 3.27 cc graft volume was inserted and filled with demineralized allogeneic bone matrix (Shanxi Aorui Medical Technology Inc, Shanxi, China).

The decision on the selection of fixation depended on the bone density. For the patient with a lower density T score less than − 2.0, posterior percutaneous fixation was performed for a stronger reconstruction. In group A (ASRSF), where an anterior single rod-screw was used (Legacy spinal system, Memphis, TN, USA), the position of the fusion cage was determined under fluoroscopy and the docking point of the screw was located at the middle part of the vertebral body. In group B (PPSF), where a posterior percutaneous screw was used (Ruizhi Medical Technology Inc, Shanghai, China), screws were inserted under biplane fluoroscopy. Both groups did not undergo posterior decompressions.

Non-steroidal anti-inflammatory drugs combined with muscle relaxants were used for post-operative analgesia. The back muscle function was exercised by swimming and gymnastics named “skydiver to superman to swimmer, “as recommended by the North American Spine Society (NASS).

### Data collection

Demographic data including gender, age, bone density (BMD), body mass index (BMI), and surgical segments were collected. The visual analog scale VAS was used to rate low back and lower extremity pain. The Oswestry dysfunction index (ODI) was used to evaluate pre- and post-operative lumbar function. Follow-ups were performed at one week, three months, 12 and 24 months after surgery.

All patients underwent routine pre-and post-operative standing anteroposterior and lateral plain and flexion–extension plain X-rays to assess inter-segmental stability, the degree of slippage and postoperative correction of slippage. Computer tomographic (CT) scans were used to evaluate the presence of cage migration or subsidence and to identify bone fusion and images were sliced two mm thick. Subsidence was defined as a cage sinking into an adjacent vertebral body by more than two mm, based on comparisons with previous CT images. Cage migration was defined as a posterior movement of the cage by three mm or more compared with the immediately post-operative image. The CT scan was also used to measure the height of intervertebral space and foramen before and after the operation. The imaging measurement was independently performed by two independent radiographers who did not participate in the study. The intra-class correlation coefficients of all variables were greater than 0.85. The height of the intervertebral space was defined as the vertical distance between the tangent lines of the upper and lower endplate dome. The height of the foramen was defined as the distance from the lower position of the upper pedicle to the upper position of the lower pedicle in the target segment. The intervertebral fusion was evaluated with the Bridwell standard, in which grades 1 and 2 were clinical fusion.

### Data analysis

Statistical analysis was performed using SPSS 18.0 for Windows (IBM, Armonk, NY, USA). Normally distributed continuous variables were presented as means ± standard deviation and were analyzed with the student’s *t* test. Non-normally distributed continuous variables were presented as medians (range) and were analyzed with the Wilcoxon test. Categorical variables were presented as frequencies and were analyzed with the Pearson Chi-Square test or the Fisher’s exact test, as appropriate. All tests were two-tailed, and *P* values < 0.05 were considered statistically significant.

## Results

Demographic data for the patients in this study are shown in Table 1. A total of 53 patients were included in this study. According to the fixation method, patients were divided into the ASRSF group A (*n* = 25) and the PPSF group B (*n* = 28). No significant difference in demographic data was established between groups (Table 1). The VAS scores of pre-operative low back pain were 5.1 ± 1.3 in group A and 5.3 ± 1.6 in group B without significant difference (*P* = 0.88). The VAS scores of leg pain were 4.5 ± 2.3 in group A and 3.9 ± 2.9 in group B and the difference was not significant (*P* = 0.65). The pre-operative ODI was 38.1 ± 4.6 in group A and 37.2 ± 3.0 in group B, with no significant difference found (*P* = 0.44) (Table 2).

**Table 1:** Comparison of patients’ demographic parameters and pre-operative imaging parameters

	ASRSF + OLIF	PPSF + OLIF	*P*
N	25	28	
**Gender**			
Male	12	17	0.42
Female	13	11	
Age	43.3 ± 7.8	47.4 ± 8.9	0.81
BMI	28.4 ± 9.0	26.7 ± 7.7	0.66
BMD	− 1.3 ± 1.2	− 1.9 ± 1.0	0.13
**Segments**			
L2,3	6	3	0.38
L3,4	10	11	
L4,5	9	14	
**Degree of slippage**			
I°	16	19	0.78
II°	9	9	
**Distribution of symptoms**			
Low back pain	4	5	0.86
Claudication	8	9	
Both	13	14	

ASRSF, anterior single rod-screw fixation; PPSF, percutaneous pedicle screw fixation

**Table 2:** Comparison of the VAS score of low back pain and ODI index before and after surgery

	ASRSF + OLIF	PPSF + OLIF	*P*
N	25	28	
**VAS**			
Pre-surgery	5.1 ± 1.3	5.3 ± 1.6	0.88
1-week post-surgery	2.3 ± 0.5	3.5 ± 0.4	0.01*
3 months post-surgery	2.2 ± 0.3	3.0 ± 0.3	0.01*
12 months post-surgery	2.2 ± 0.8	2.4 ± 0.8	0.34
24 months post-surgery	2.3 ± 0.6	2.3 ± 0.7	0.87
**ODI**			
Pre-surgery	38.1 ± 4.6	37.2 ± 3.0	0.44
1-week post-surgery	25.1 ± 4.6	27.2 ± 6.0	0.47
3 months post-surgery	17.0 ± 5.2	17.9 ± 6.3	0.58
12 months post-surgery	19.3 ± 5.6	20.4 ± 7.5	0.55
24 months post-surgery	12.65 ± 3.6	19.1 ± 3.4	0.01*

*The difference was statistically significant.

ASRSF, anterior single rod-screw fixation; PPSF, percutaneous pedicle screw fixation

No significant difference was found in pathological segment distribution (*P* = 0.38) or the degree of slippage (*P* = 0.78) between the two groups (Table 1). No significant difference was found between the two groups in symptom distribution (*P* = 0.86). The pre-operative lumbar lordosis was 34.6° ± 4.1° in group A and 35.5° ± 3.8° in group B, which was not significantly different (*P* = 0.41). The pre-operative intervertebral space height was 10.8 ± 2.4 mm in group A and 10.3 ± 2.1 mm in group B without significant difference (*P* = 0.46). The height of the pre-operative foramen was 12.5 ± 1.6 mm in group A and 12.5 ± 1.4 mm in group B, also without significant difference (*P* = 0.92).

The post-operative clinical results were also compared. The VAS score of leg pain at three months post-surgery was 1.1 ± 0.7 in group A and 1.3 ± 0.8 in group B, with no significant difference (*P* = 0.71). One week after surgery, the VAS score of low back pain in group A was significantly lower than in group B (2.3 ± 0.5 vs 3.5 ± 0.4, *P* = 0.01). At three months post-surgery, the reported low back pain in group A was still significantly lower than in group B (2.2 ± 0.3 vs 3.0 ± 0.3, *P* = 0.01). The superiority of group A in reducing low back pain disappeared at 12 months post- surgery (Table 2). At 24 months post-surgery, the post-operative ODI was 12.65 ± 3.6 in group A and 19.1 ± 3.4 in group B, which was significantly different (*P* = 0.01). There was no significant difference in the ODI between groups at one week, three months and 12 months post-surgery.

Three months post-surgery, the foramen height was 18.1 ± 2.3 mm in group A and 16.9 ± 1.9 mm in group B, showing a statistical difference (*P* = 0.04). Twelve months after the operation, there was no significant difference concerning the change in foramen height. No statistical difference was found in the height of the intervertebral space between the two groups at any follow-up time (Table 3). No statistical difference was found in the segmental lordosis correction or slippage correction between the two groups at 12 months post-surgery (Table 3).

**Table 3:** Comparison of imaging parameters between the two groups

	ASRSF + OLIF	PPSF + OLIF	*P*
N	25	28	
**Intervertebral space height**			
Pre-surgery	10.8 ± 2.4 mm	10.3 ± 2.1 mm	0.46
3 months post-surgery	14.1 ± 1.0 mm	14.5 ± 0.9 mm	0.12
12 months post-surgery	14.0 ± 1.0 mm	14.3 ± 1.0 mm	0.38
**Foraminal height**			
Pre-surgery	12.5 ± 1.6 mm	12.5 ± 1.4 mm	0.92
3 months post-surgery	18.1 ± 2.3 mm	16.9 ± 1.9 mm	0.04*
12 months post-surgery	16.9 ± 2.1 mm	16.6 ± 2.3 mm	0.60
**Lumbar lordosis**			
Pre-surgery	34.6° ± 4.1°	35.5° ± 3.8°	0.41
3 months post-surgery	45.7° ± 5.6°	45.7° ± 6.3°	0.97
12 months post-surgery	45.2° ± 5.6°	44.2° ± 3.6°	0.44
**Segmental lordosis**			
Pre-surgery	5.4° ± 2.1°	6.1° ± 2.4°	0.34
12 months post-surgery	10.1° ± 2.7°	12.8° ± 3.0°	0.46
Slippage correction at 12 months	0.92 ± 0.07	0.94 ± 0.1	0.84

ASRSF, anterior single rod-screw fixation; PPSF, percutaneous pedicle screw fixation

*The difference was statistically significant

There was a significant difference between the pre- and 12 months post-surgery imaging parameters between the two groups (Table 4). The Bridwell method was applied in the fusion assessment with grade I and II considered as successful fusion. Twelve months after the operation, 18 cases (72%) achieved fusion in group A and 22 (78.6%) in group B. There was no significant difference between the two groups (χ^2^ = 0.31, *P* = 0.75). All patients achieved fusion at 24 months post-surgery. No subsidence was found in any patients during follow-ups.

**Table 4:** Comparison of imaging parameters before and 12 months after surgery

	ASRSF + OLIF	*P*	PPSF + OLIF	*P*
N	25		28	
**Intervertebral space height**				
Pre-surgery	10.8 ± 2.4 mm		10.3 ± 2.1 mm	
12 months post-surgery	14.0 ± 1.0 mm	0.02*	14.3 ± 1.0 mm	0.01*
**Foraminal height**				
Pre-surgery	12.5 ± 1.6 mm		12.5 ± 1.4 mm	
12 months post-surgery	16.9 ± 2.1 mm	0.01*	16.6 ± 2.3 mm	0.01*
**Lumbar lordosis**				
Pre-surgery	34.6° ± 4.1°		35.5° ± 3.8°	
12 months post-surgery	45.2° ± 5.6°	0.03*	44.2° ± 3.6°	0.03*

ASRSF, anterior single rod-screw fixation; PPSF, percutaneous pedicle screw fixation

*Comparing with pre-operative imaging parameters, the difference was statistically significant

**Fig. 1:**
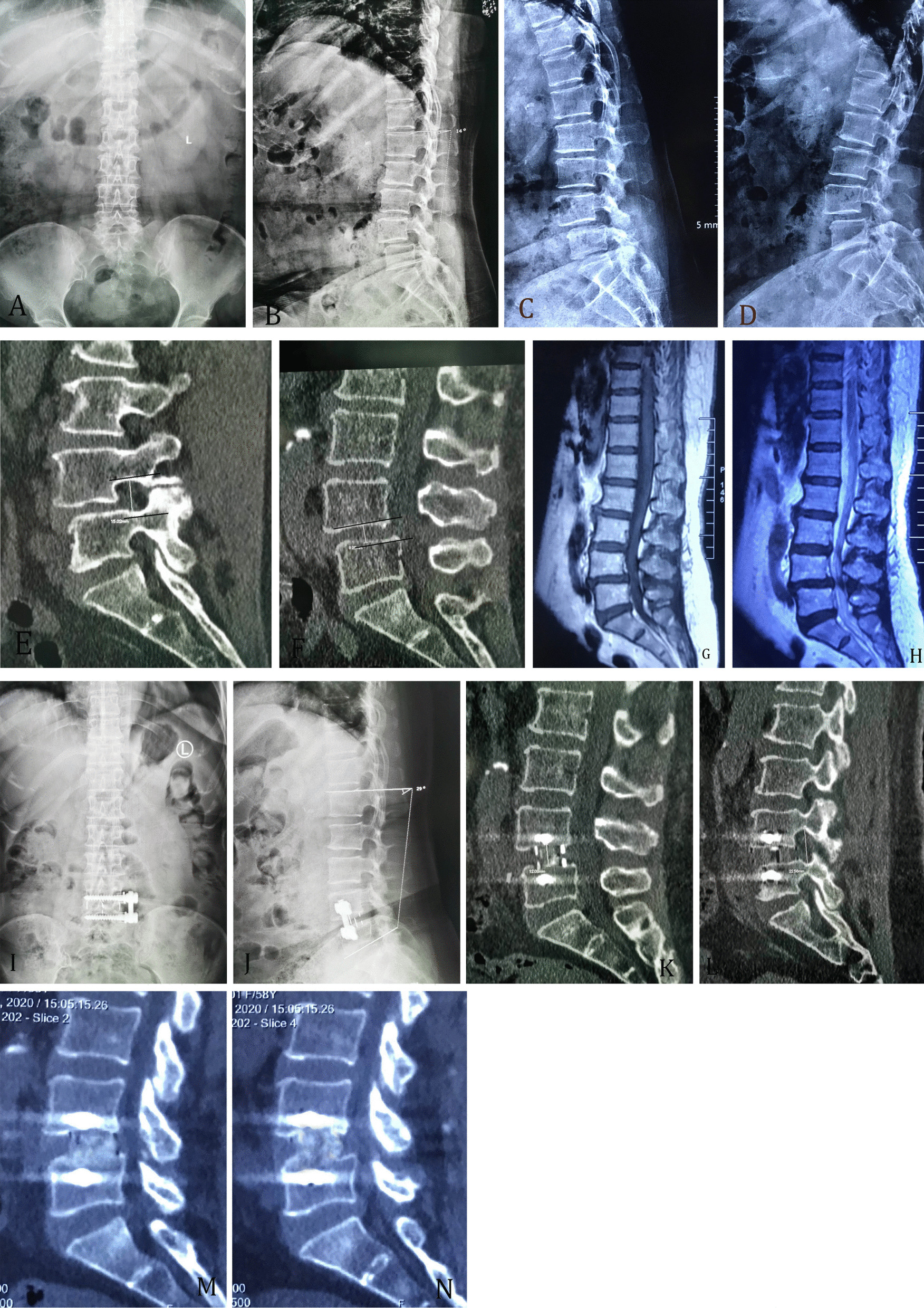
A typical case. **A**–**D** Pre-operative radiographies with lordosis of 14°; **E** height of intervertebral foramen before surgery; **F** height of intervertebral space before surgery; **G** pre-operative MRI T1 weighted imaging H,pre-operative MRI T2 weighted imaging; **I**, **J** post-operative radiographies with lordosis of 29°; **K** post-operative intervertebral space height; **L** post-operative intervertebral foramina height; **M** 12 months post-surgery,solid fusion at upper endplate of L4. **N** 24 months post-surgery solid fusion at the upper and lower endplates

In terms of complications, one patient in group A encountered a limited cage migration at three months post-surgery. There were four cases of post-operative abdominal distension, two cases of dysuria, and two cases of transient numbness at the anterolateral portion of the thigh in group A. No instrument complication occurred. In group B, there was one case of posterior subcutaneous hematoma, two cases of screw misplacement without neurological complication, one case of superficial skin infection, three cases of abdominal distension and one case of transient thigh numbness. There were no statistical differences in complications between the two groups (χ^2^ = 4.71, *P* = 0.31). A typical case is shown in Fig. 1.

## Discussion

Due to the advantages of reducing soft tissue damage, improving lumbar lordosis, and reducing nerve tissue disturbance, OLIF has attracted recent attention from spine surgeons. It has been found to increase the cross-sectional area of the dural sac by a median of 30.2% and increase the neural foramen area by an average of 30.0% [11–14]. Compared with traditional posterior TLIF surgery, the probability of nerve root injury was about 1.3% using OLIF [12, 14]. No patients in this study experienced post-operative nerve root edema or direct nerve root injury, which indicated that OLIF was a superior technique by better protecting nerve tissue.

Since OLIF does not involve iatrogenic damage to the posterior structure, the impairment of spinal stability is limited. There has been controversy about whether additional fixation is necessary [6, 14]. Due to the different elasticity modulus between the cage and the endplate, there is a risk of subsidence when the cage is used alone [14, 15], so the insertion of pedicle screws is necessary for patients with endplate damage, osteoporosis, or post-operative residual radicular symptoms that require posterior surgeries [13, 15]. Lin et al. [16] evaluated 52 patients who underwent OLIF without posterior instrumentation and reported a fusion rate of 81.9% at 24 months after surgery as assessed by CT scan imaging. Kim et al. [17] reported a 12 month fusion rate of 92.9% in 29 OLIF patients with posterior pedicle screw fixation as assessed with CT. In the present study, no cage subsidence nor nonfusion were found within 24 months post-surgery, which further confirmed that limited internal fixation can reduce the risk of cage subsidence and promote intervertebral space fusion.

In this study, the intervertebral space fusion rate at 12 months post-surgery was 72% in group A and 78.6% in group B, which were low. The major negative factor for the inferior fusion rate was likely to be the material of the bone graft. Compared with iliac crest or bone morphogenic protein, using allogeneic bone could result in a lower fusion rate [11, 13].

Posterior percutaneous screw fixation could reduce paraspinal muscle damage where iatrogenic impairment to the paravertebral soft tissues was unavoidable, but this could lead to muscular atrophy and low back pain. Also given the lack of anatomical reference markers, percutaneous pedicle screw implantation has a higher incidence of screw misplacement compared with open surgery [7, 8]. Using a corridor by OLIF for segmental fixation can effectively reduce the risk of screw misplacement for the direct procedure of implantation and the massive docking area for screws. In this study, when compared with PPSF, ASRSF resulted in lower back pain at one week and three months after surgery. The ODI index was also lower in the ASRSF group at 24 months post-surgery, which confirmed the superiority of ASRSF in relieving pain and improving lumbar function. This may be due to evidence of paraspinal muscle protection.

The height of the intervertebral foramen was better maintained at three months post-surgery in the ASRSF group compared with the PPSF group. It was hypothesized this might originate from the close position to the central axis of the spine in the anterior fixation group, which diminishes the ‘self-locking’ phenomenon in the posterior fixation that could result in a reduction of the intervertebral foramen area [18]. However, this superiority disappeared at 12 months post-surgery, which might be related to an increase in lumbar lordosis. Posterior fixation could result in better reconstruction of segmental lordosis for the same reason, but there was no significant difference in the comparison of segmental lordosis correction between two groups in this study, which could be a bias due to the small sample.

The use of ASRSF showed good stability in the debridement of intervertebral space and anterior vertebrectomy, which confirmed that the single screw rod could provide stability if the posterior column were intact [19–21]. In this study, there was no internal fixation migration in the anterior group, supporting the stability of anterior fixation solely. This was contributed by the inaction of the posterior column in the OLIF procedure and the average age of the patients in this study of 43.3 years. The pre-operative BMD was around − 1.3, which equally contributed to maintaining stability and the excellent skeletal condition of the participants. Patients with lumbar isthmic spondylolisthesis were excluded from this study in considering the superiority of the posterior approach, as extra dissection at the isthmus was necessary for a better reduction.

In summary, ASRSF combined with OLIF for lumbar spondylolistheses reduced early post-operative low back pain more effectively, increased the area of foramen and improved post-surgery lumbar function compared with the clinical manifestation of PPSF with OLIF. In addition, it provided sufficient stability for intervertebral space fusion.

This study has limitations. Firstly, this study was a retrospective one where the corresponding author performed all the operations, so due to the lack of case selection, there might be a selection bias. For the selection of fixation methods, empiricism might post negative influence on decision-making and to enhance the efficiency of communication between surgeons and patients, three-dimensional printing is strongly recommended for visualization of the procedure [22]. Secondly, this study was a single-center study with a small sample size, therefore it was impossible to evaluate independently based on surgical segments. Thirdly, since patients in this study were young with ideal bone quality, further research is needed on whether the conclusions made here are appropriate for elderly patients. Finally, it should be pointed out the major factor affecting the iatrogenic damage is the choice of surgical approach. The influence of fixation methods on the patient`s postoperative functional parameter is smaller. Large-scale randomized controlled studies are needed to draw definite conclusions.

## Data Availability

All data analyzed during this study are included in this published article.

## Abbreviations

ASRSF: Anterior single rod-screw fixation
PPSF: Percutaneous pedicle screw fixation
DSD: Degenerative spinal disease
DLS: Degenerative lumbar spondylolisthesis
